# FLT3-Mutated Leukemic Stem Cells: Mechanisms of Resistance and New Therapeutic Targets

**DOI:** 10.3390/cancers16101819

**Published:** 2024-05-10

**Authors:** Debora Capelli

**Affiliations:** Department of Hematology, Azienda Ospedaliera Universitaria, Ospedali Riuniti di Ancona, Via Conca 71, 60126 Ancona, Italy; debora.capelli@ospedaliriuniti.marche.it; Tel.: +39-071-5963930

**Keywords:** FLT3-mutated AML, FLT3 inhibitors, leukemic stem cell metabolism, leukemic stem cell mechanisms of resistance, leukemic stem cells molecular pathways, CAR-T, CAR-NK, immunotherapy

## Abstract

**Simple Summary:**

The treatment and cure of FLT3-mutated AML (FLT3mut) is still a challenge despite the availability of mutation-targeted FLT3 inhibitors (FLT3i). This is a review of possible mechanisms of leukemic stem cell (LSC) resistance focusing on immunophenotype, metabolism, molecular pathways of activation, and interaction with stroma in FLT3mut AML. A comprehensive review of possible new targets and treatments is reported for each section, with emphasis on the metabolism of LSC, their molecular pattern of activation, and immunotherapy.

**Abstract:**

Despite the availability of target drugs in the first and second line, only 30% of FLT3mut AMLs are cured. Among the multiple mechanisms of resistance, those of FLT3mut LSC are the most difficult to eradicate because of their metabolic and genomic characteristics. Reactivation of glycogen synthesis, inhibition of the RAS/MAPK pathway, and degradation of FLT3 may be potential aids to fight the resistance of LSC to FLT3i. LSC is also characterized by the expression of a CD34+/CD25+/CD123+/CD99+ immunophenotype. The receptor and ligand of FLT3, the natural killer group 2 member D ligand (NKGD2L), and CD123 are some of the targets of chimeric antigen receptor T cells (CAR-T), bispecific T-cell engager molecules (BiTEs), CAR-NK and nanoparticles recently designed and reported here. The combination of these new therapeutic options, hopefully in a minimal residual disease (MRD)-driven approach, could provide the future answer to the challenge of treating FLT3mut AML.

## 1. Introduction

The target drugs Midostaurin and Gilteritinib have been approved for the treatment of FLT3mut AML, respectively, due to the results achieved in phase III trials. Midostaurin and 3+7 showed a median OS of 74.7 months, but 40% of patients were refractory to Midostaurin, and 50% of responders relapsed due to the emergence of various primary or secondary resistance mechanisms [1]. Relapsed or refractory patients treated with Gilteritinib achieved a median OS of 9.3 months, with a CR rate of 34%, 2-year OS of 20.6%, and 75.7% 2-year cumulative incidence of relapse at the median survival follow-up of 37.1 months [2].

FLT3i also plays a role in cancer treatment. Sorafenib and Cabozantinib are indicated in the treatment of hepatocellular and advanced renal cell carcinoma, and Cabozantinib is also indicated in the treatment of differentiated thyroid carcinoma locally advanced or metastatic, refractory or ineligible for radioactive iodine therapy.

Hasegawa et al. showed that 85 patients with metastatic colon cancer and FLT3 amplification had a shorter median survival than those without such amplification [3].

Conversely, FLT3 expression in breast tumors has a positive prognostic significance, with improved survival. The correlation between the expression of FLT3 and the presence of a greater number of immune cells, such as CD4+ T, CD8+ T, myeloid dendritic cells, and neutrophils in the tumor tissue and the association with a reduced burden of tumor mutations suggests the rationale for the positive predictive significance of FLT3 in tumor prognosis and for a possible use of FLT3L as immunotherapy [4].

A similar role of FLT3 was shown in patients affected by pancreatic ductal adenocarcinoma. FLT3 and PCBP3, an inhibitor of DNA and RNA transcription, have been identified as potential biomarkers for a favorable prognosis of pancreatic cancer [5].

In a previous review, we showed that several next-generation sequencing analyses of samples from patients treated with type I and type II FLT3i identified mutations in the RAS/MAPK pathway, among the most frequently associated with primary and secondary resistance mechanisms in AML [6]. Relapses can be caused by LSC already present at diagnosis or by more committed cells. In some cases, recurrences arise from the preleukemic clone due to the proliferative advantage conferred by mutations such as DNMT3A or to the pro-inflammatory phenotype associated with the TET2 mutation [7]. Resistance to FLT3mut LSC is the most difficult to eradicate and is the real pitfall of curing FLT3mut AMLs. Patients affected by FLT3-ITD pos AML with high allelic ratio showed to express higher levels of CD69 and a high percentage of CD69 hematopoietic stem cell (HSC)-like cells demonstrated to be related to MRD, low OS, and high relapse incidence [8].

Zhang et al. analyzed cells from pediatric AML patients before and after intensive chemotherapy, demonstrating that a small fraction of CD69 HSC-like cells present at diagnosis possessed the LSC signature and could persist after intensive chemotherapy, arguing a possible correlation between senescence and chemotherapy resistance. Indeed, CD69 was associated with decreased total protein levels of mTOR and P70S6K in HL60 and Kasumi-1 cell lines, resulting in reduced proliferation and maintenance of senescence. The authors also demonstrated the correlation between CD69 and reduced expression of the proliferation marker Ki67 and of the regulators CCND1 and CDK6, and with the expression of the adhesion molecule CXCR4 promoting the binding of the leukemic cell to the stromal niche receptor CXCL12, highlighting the role of senescence and adhesion as possible mechanisms of evasion of the antileukemic action of chemotherapy. Chemoresistance was also associated with activation of the metabolism OXPHOS in leukemic progenitor cells (LMPP-like and GMP-like) as demonstrated in a patient-derived xenograft (PDX) model treated with cytarabine [9]. Understanding the mechanisms that determine the proliferation and quiescence of LSC is critical for the identification of new targets for the eradication of FLT3mut LSC.

## 2. Results

### 2.1. Mechanisms of Resistance of FLT3mut LSC

Successful treatment of FLT3mut AML requires an efficient targeting of LSC but FLT3i can fail to eradicate these cells and thus result in relapse.

The analysis of immunophenotype, metabolism, and gene and RNA expressions of LSC has identified some interesting targets for the development of new therapeutic options that can overcome resistance mechanisms. As previously described [3] authors have identified the acquisition of new mutations as secondary mechanisms of resistance and the presence of some primary pan-resistant FLT3 mutations (F691L) after FLT3i treatment. We focused our review on the analysis of LSC resistance, assuming that some of the relapses are due to the persistence of LSC. Bone marrow niche, amino acids, and fatty acid metabolism, and the gene expression profile of FLT3mut LSC are responsible for their immunological escape, survival, and dormancy with consequent resistance to FLT3i, Venetoclax, and chemotherapy. In the follow-up after treatment, FLT3mut LSC might therefore lose their stemness due to metabolic or acquired molecular mutations and generate more committed leukemic progenitors with proliferative advantage resulting in relapse. Here, we report all the targets found to be involved in the resistance of FLT3mut LSC to AML treatments.

### 2.2. Immunophenotype of FLT3mut LSC

Murine models of FLT3-ITDmut AML have shown that LSC also expresses the mutation despite the fact that this mutation is a late event. Angelini et al. showed how FLT3ITDmut AML cells express the CD34+/CD25+/CD123+/CD99+ phenotype, identifying CD99 and CD123 as possible targets for immunotherapy [10]. CD123 is the α-chain of the IL3 receptor, and CD99 is the MIC-2, an O-glycosylated transmembrane antigen, involved in the regulation of apoptosis and in adhesive properties of T cells, both expressed, at high levels, in FLT3mut leukemic precursor reservoir [11]. The development of immunotherapies targeting these antigens might represent an interesting tool in LSC eradication, even in MRD-positive settings. The lack of the expression of CD99+ and low positivity of CD123 antigens in normal CD34+ hemopoietic progenitor cells might also contribute to a specific LSC killing with low hematological toxicity.

### 2.3. LSC Metabolism of FLT3mut LSC

**Figure 1:**
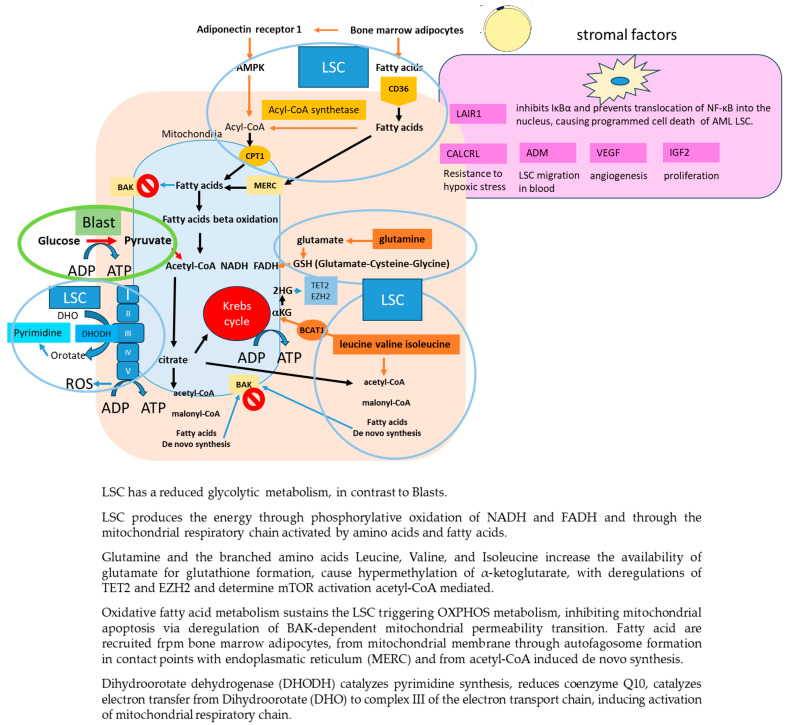
LSC metabolism and interaction with stroma.

The LSC is generally quiescent, but when it comes to activity, it has a reduced glycolytic metabolism, a low level of reactive oxygen species, promoted by hypoxia of the stromal niche, whereby it produces the energy necessary for its proliferation either through phosphorylative oxidation of NADH and FADH (NOX), provided by glutathione, which can simultaneously control reactive oxygen species production, or through the mitochondrial respiratory chain activated by amino acids and fatty acids [12]. This alternation of metabolism is able to trigger resistance to traditional drugs but also to inhibitors of phosphorylative oxidation of NADH and FADH, such as Venetoclax. Figure 1 shows the metabolism of LSC. The branched amino acids Leucine, Valine, and Isoleucine increase the availability of glutamate for glutathione formation (glutamate, cysteine, and glycine) through the transfer of amino acid groups to α-ketoglutarate by branched amino acids aminotransferase 1 and cause hypermethylation of α-ketoglutarate, with deregulations of *TET2* and *EZH2* similar to those observed in mutated IDH1/2 cells. Leucine converted to acetyl-CoA also activates mTOR complex 1, promoting LSC survival.

Oxidative fatty acid metabolism also sustains the LSC, especially in relapsed patients. The mitochondria support LSC by activating autophagy, by forming autophagosomes at the contact sites between the endoplasmic reticulum and mitochondrial membrane, that degrade lipid droplets by releasing fatty acids, ready to trigger OXPHOS metabolism [13]. Fatty acids also inhibit mitochondrial apoptosis via deregulation of BAK-dependent mitochondrial permeability transition, inducing resistance to chemotherapeutic drugs.

Glycogen synthase kinase 3 (GSK3), is also an important key regulator of the LSC metabolism inhibiting glycogen synthase and promoting protein synthesis and glycolysis. It also has an ambiguous role as a tumor suppressor due to the degradation inactivation of *B-catenin-MCL1-MYC* and activation of p53. Recent studies have shown that FLT3 ITD mutation can trigger leukemogenesis through activation of GSK3 and its induced changes in LSC metabolism, making this enzyme a possible therapeutic target [14].

LSC survival relies on the synthesis of pyramidines, the inhibition of which can cause their death. The enzyme dihydroorotate dehydrogenase (DHODH), located in the inner mitochondrial membrane, is able to catalyze pyrimidine synthesis. Inhibition of DHODH and glutamine metabolism has been shown to reduce LSC and leukemic cell burden in PDX mouse models [15,16]. Pyrimidine de novo biosynthesis is closely related to abnormal cell proliferation.

Inhibition of DHODH has been shown to promote myeloid differentiation in a GMP ER-*HoxA9* cell line derived from mouse bone marrow knocking for lysozyme-GFP [12]. Cell line differentiation was induced by DHODH inhibitors through inhibition of the downstream enzyme ‘Orotydilate decarboxylase’ and subsequent blockade of uridine monophosphate (UMP) synthesis, independent from the expression of Dihydroorotate which hence does not represent a biomarker of leukemogenesis, as 2-hydroxyglutarate (2HG) is in patients with IDH mutated leukemias. Brequinar sodium, a DHODH inhibitor, reduced leukemic growth in a PDX model of FLT3-ITD AML.

Furthermore, DHODH reduces coenzyme Q10 and catalyzes the electron transfer from Dihydroorotate (DHO) to complex III of the electron transport chain, activating the mitochondrial respiratory chain [17].

DHODH inhibitors also affect protein transduction via inhibition of the downstream pathway controlled by the enzyme O–N-acetylglucosamine transferase, which catalyzes the N-acetylglycosylation of proteins such as Akt, TET, and c-Myc by transferring N-acetylglucosamine from UDP- N-acetylglucosamine to serine and threonine residues of these proteins [18]. DHODH inhibitors also inhibit mTOR promoting autophagy and degradation of FLT3 protein, demonstrating synergy with Quizartinib in mouse models of FLT3mut AML [19].

### 2.4. Gene Expression of FLT3mut LSC

**Figure 2:**
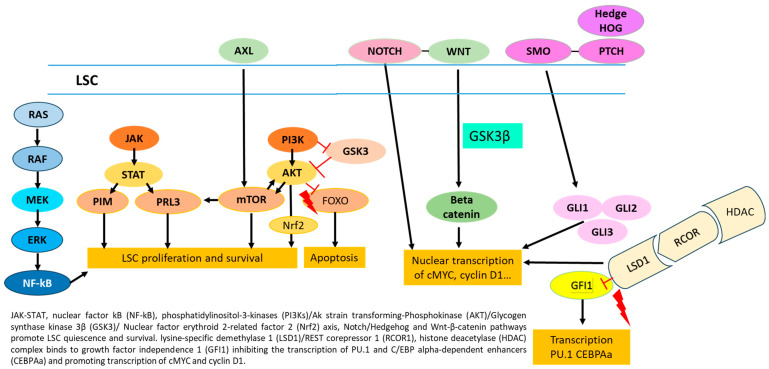
Molecular pathways promoting stemness and survival of LSC.

A recent study showed specific activation of *mTOR, p-4EBP1*, and *p-S6* in LSC, compared with normal progenitors, identifying this pathway as a possible target for therapy [20]. *JAK-STAT, NF-kB*, PI3K/Akt/Nrf2, *Notch*, *Hedgehog*, and *Wnt-β-catenin* pathways are all able to promote leukemia cell stemness and survival (Figure 2). Chemotherapy resistance of LSC is also mediated by the expression of genes, including *MYC*, transcription factors, such as *EVI1*, and epigenetic modifications induced by *HIF1α* and other factors, which regulate metabolic properties, plasticity, and the transition of LSC from quiescence to cell proliferation [21]. NF-κB activation enables LSC to evade apoptosis. *Wnt/β-catenin* and *Hedgehog* play important roles in the regulation of self-renewal of LSC and normal HSCs through the expression of target genes such as *c-Myc, c-Jun*, and *cyclin D1* [22].

Dysregulation of *JAK/STAT* and/or PI3K/AKT/mTOR contributes to the maintenance of LSC, representing a possible target in LSC eradication (Figure 2). The LSC also overexpresses lysine-specific demethylase 1 (LSD1), capable of blocking the action of BET inhibitors on myc expression through its action of scaffolding the *c-MYC*/HBXIP/Hotair/LSD1 complex determining the transcription of *c-MYC* genes [23]. It also promotes the maintenance of stemness through the binding of the LSD1/RCOR1 complex to GFI1. Indeed, LSD1 and the corepressor RCOR1 regulate stem cell differentiation by binding GFI1 and inhibiting the transcription of PU.1 and *C/EBP alpha-dependent enhancers* and their target genes involved in LSC differentiation (Figure 2).

### 2.5. Interaction of FLT3mut LSC with Stroma

Among the stromal factors regulating LSC homing and chemoresistance, adrenomedullin increases leukemic cell migration through the fenestrated endothelium of the marrow; its Calcitonin receptor-like receptor promotes LSC resistance to leukemic blast growth stress; vascular endothelial growth factor promotes angiogenesis; and insulin-like growth factor 2 regulates LSC proliferation via the IGF2/IGF1R/Nanog pathway [24,25].

Leukocyte-associated immunoglobulin-like receptor-1 (LAIR-1) is a transmembrane glycoprotein with a single immunoglobulin-like domain and a cytoplasmic tail containing two immune receptor tyrosine-based inhibitory motifs. It is expressed by myeloid, T, and B cells, and its binding to collagen induces receptor clustering and phosphorylation of their immune receptor tyrosine-based inhibitory motifs with subsequent activation of downstream inhibitory immune modulation through phosphorylation of phosphatase-1. Activation of LAIR-1 receptors on LSC inhibits IκBα and prevents translocation of NF-κB into the nucleus, causing programmed cell death [26].

Activation of LAIR-1 stimulates proliferative signals, such as CAMK1/CREB, counteracted and nullified, however, by suppression of downstream mediators such as NF-κB, MAPK, and Src kinase, resulting in programmed cell death of AML cells. Stimulation of LAIR-1 does not promote apoptosis in healthy stem cells, compared with leukemic cells, possibly due to different mTOR/AKT/NF-κB activity, but the specific mechanism of action will be the subject of future studies [27].

Hypoxia of the LSC niche also promotes the release of HIF1a, which upregulates the expression of CXCR4 by LSC and their adhesion to stroma and transendothelial migration mediated by Stromal Cell-Derived Factor-1; HIF1a also activates LSC genes responsible of their dormancy [28]. Figure 1 shows the interaction of LSC with stroma.

### 2.6. New Approaches to Treatment of FLT3mut LSC Resistance

We will then analyze drugs that can inhibit FLT3mut LSC growth, including those that can suppress its metabolism, activation pathways, the RAS-MAPK pathway, interaction with stroma, and immunotherapy directed against the LSC.

GSK3 inhibits glycogen production and promotes protein synthesis and glycolysis, resulting in increased pyruvate production [11]. The GSK3 inhibitor, BIO, confirmed the growth inhibition of FLT3ITDmut MV4-11 cells both in in vitro cultures and in in vivo xenograft models in BALB/c mice, exactly through the arrest of glycolysis due to the suppression of enolase 1 and GSK3. Indeed, metabolomic analysis of MV4-11 cell cultures treated with the GSK3 inhibitor BIO showed a reduction in pyruvate and ATP production due to glycolysis blockade. BIO also results in cell arrest in G1 due to the increase of cyclin D2 and inhibition of p21; it also induces apoptosis of leukemic cells via activation of the caspase 3 pathway.

Among DHODH inhibitors, Brequinar has been administered in Phase I/II clinical trials to patients with relapsed/refractory AML, the results of which are awaited (NCT03760666). Brequinar has been ineffective in several clinical trials involving solid tumors due likely to insufficient inhibition of pyrimidine synthesis, likely related to the cellular permeability of the drug. The supply of Uridine transported from the extra to the intracellular compartment via the channels of the human nucleoside equilibrative transporter may also bypass DHODH-induced inhibition of pyrimidine synthesis.

Among the DHODH inhibitors, MEDS433 and compound 4, with a decorated aryloxyaryl moiety in place of the biphenyl scaffold, showed differentiating effects on AML THP1 cells, which were superior to those of brequinar and enhanced by dipyridamole, via inhibition of pyrimidine supply, due to blockade of hENT1/2. Compound 4 showed significant antileukemic activity in vivo in a xenografted mouse model of AML [29].

So et al. showed that the novel DHODH inhibitor AG636 led to the inhibition of de novo pyrimidine synthesis [30]. CDK5 was identified, in a CRISPR-Cas9 knockout screen, as a sensitizer to DHODH inhibition, suggesting a possible simultaneous target of different mitochondrial processes.

Phase I studies with PTC299 (Emvododstat NCT03761069) and with JNJ-74856665, alone or in combination with Azacitidine or Venetoclax (NCT04609826), are now active in refractory AML patients but are not recruiting patients.

Inhibitors of LSD1, BTK, AKT/mTOR/S6K, and MAPK have achieved results in in vitro and in vivo studies via inhibition of LSC activation pathways. LSD1 inhibitors suppress transcriptional regulatory factors of STAT5 and C-MYC target genes via methylation of Hystone 3 Lysin 9 (H3K9) and Hystone 3 Lysin 4 protein. They can thus be synergistic with FLT3i due to the repressive methylation of H3K9me1, a substrate of LSD1, which prevents its binding to the superenhancer MYC [23].

The LSD1 inhibitor tranylcypromine, in combination with ATRA and cytarabine, is currently being evaluated in a phase I study (NCT02717884). Iadademstat (ORY-1001) demonstrated efficacy and good tolerability at the dosage of 140 mg/sm/d in the 14 patients in the expansion cohort of the phase I study EudraCT 2013-002447-29 [31]. The phase I study with the all-oral combination Gilteritinib-Iadademstat (ORY-1001) is currently enrolling patients (NCT05546580).

In vitro studies have shown that LSD1 inhibitors are synergistic with those of BCL-2 and MDM2, Venetoclax, and RG7388 by increasing pro-apoptotic proteins [32]. Bomedemstat (IMG-7289)-Venetoclax, another all-oral combination, is being investigated in a phase I study (NCT05597306). Results of the recently completed phase I study with Bomedemstat +/− ATRA, NCT02842827, are awaited. In contrast, the phase I study NCT02177812 with the inhibitor GSK-2879552 was stopped early due to lack of benefit.

Autophagy represents one of the mechanisms of resistance to FLT3i. Bruton tyrosin kinase (BTK) inhibitors are capable of suppressing BTK-related autophagy. The FLT3/BTK/Aurora kinase *(AURKs)* inhibitor luxeptinib (CG-806), due to its multi-target action, causes the arrest of blasts in G2/M, promotes polyploidy formation, stimulates apoptosis, and inhibits BTK-mediated autophagy, by-passing resistance to FLT3i [33]. Yu et al. [34] confirmed these data in immunoblotting analyses of CG-806-treated cell lines. In fact, inhibition of AKT/mTOR/S6K and MAPK results in suppression of p-mTOR, -S6K, and -RB cell proliferation proteins, while inhibition of BTK causes upregulation of p27 and downregulation of G1 phase checkpoint proteins, such as CDK4, CDK6, and c-Myc, blocking FLT3mut cells (MOLM3 and MV14-4) in G1. Inhibition of AURK and Polo-like kinase 1 causes reduced levels of p-AURK B and C, PLK1, p-CDC25c, and cyclin B1, resulting in the arrest of FLT3 WT cells in G2/M (THP-1). The authors demonstrated a synergy between CG-806 and inhibitors of Bcl-2 (venetoclax) and/or Mcl-1 (A1210477) that can increase apoptosis independently of FLT3 mutation expression, even in the presence of the pan-resistant “gatekeeper” mutation F691L. CG-806 alone showed reduced efficacy on Mcl-1, but combination with venetoclax and/or A1210477 resulted in activation of the intrinsic apoptosis pathway, increasing the level of caspase-3 cleavage. A phase 1 study is currently ongoing in FLT3mut R/R LAMs (NCT04477291).

CG-806 alone showed reduced efficacy on Mcl-1, but the combination with venetoclax and/or A1210477 resulted in activation of the intrinsic apoptosis pathway, increasing the level of caspase-3 cleavage. A phase 1 study is currently underway in FLT3mut R/R LAMs (NCT04477291).

ERK activation and RAS mutations are among the mechanisms of resistance to both FLT3i and BCL2 inhibitors.

The receptor tyrosine kinase, Anexelekto (AXL), is activated by FLT3ITD mutations and is responsible for resistance to Midostaurin and Quizartinib [35]. Gilteritinib targets AXL but can induce drug resistance by activating RAS mutations. ONO-7475 is a specific inhibitor of AXL and MER tyrosine kinase that reduces phosphorylation of ERK1/2 and MCL-1 expression by simultaneously inhibiting BCL2 and FLT3 signaling pathways, bypassing resistance to other Tyrosine kinase inhibitors. Post SM et al. analyzed the efficacy of the ONO-7475/ABT-199 combination using in vitro (MOLM-13) and in vivo models of FLT3ITDmut AML-PDX [36]. ONO-7475 acts both on the RAS-AXL and BCL2-RAF axis, inhibiting cells overexpressing MCL-1, improving the survival of mice transplanted with multiresistant FLT3ITDmut AML cells, compared with ABT-199 alone. The two drugs are synergistic with each other. XZB-0004 is a novel and potent small molecule inhibitor of receptor tyrosine kinase AXL currently being tested in a phase I trial in R/R patients with AML and MDS (NCT05740917).

The MEK1/MEK2 inhibitor Trametinib has shown efficacy in mono-administration with a 20% ORR in patients with AML R/R [37], but combination studies with azacitidine and venetoclax (NCT04487106), with the MDM2i AMG232 (NCT02016729) have not shown improved responses, despite the rationale for synergism.

Studies now completed with the Plk/PI3K pathways inhibitor Rigosertib (NCT00854646, NCT01167166) are awaiting results, while the sequential combination of its oral formulation after azacitidine for 21 days (NCT01926587) showed two responses in seven patients with R/R AML.

FLT3 degradation represents another potential approach to overcome drug resistance of FLT3mut AML. Bortezomib inhibits PI3K/AKT, STAT5, and MAPK/ERK and degrades FLT3 via proteasome inhibition [38]. 

A phase II study (NCT01174888) showed that Bortezomib in combination with Midostaurin and MEC (Mitoxantrone, Cytarabin, and Etoposide) chemotherapy results in 56.5% CR and 82.5% ORR in R/R AML, counting diarrhea and peripheral neuropathy among the most common adverse events [39]. Bortezomib at days 2, 5, 9, and 12, combined with Azacitidine, resulted in 5 ORRs in 23 patients with R/R AML in a phase I study (NCT00624936). The studies of Bortezomib in combination with Lenalidomide in phase I (NCT02312102), and HD-ARAC and Gemtuzumab Ozogamicin (NCT04173585) have ended, and we are awaiting results.

HSP90 and USP10 are responsible for the deubiquitination of FLT3 and the subsequent blockade of its degradation. Arsenic inhibits USP10 and has synergistic effects with Sorafenib and quizartinib, inhibiting the FLT3/STAT5/AKT/ERK pathway, and with ATRA, inhibiting C-MYC [40]. We are awaiting the results of Arsenic in the phase II study in combination with Decitabine (NCT02190695) and in the phase I study in combination with stepped doses of Cyclophosphamide (NCT03318016).

Wu5, an inhibitor of USP10 [41], and QL47 [42], an inhibitor of HSP70, induced FLT3-ITD degradation even in the presence of drug-resistant mutations, suggesting possible future use in clinical trials.

The interaction of LSC with the stroma is responsible for their maintenance of stemness, survival, and immunological escape. Lovewell et al. [27] tested in vitro the LAIR-1 agonist monoclonal antibody, NC525, which can bind human, but not murine, LAIR-1 protein by inhibiting its binding to collagen. The antibody significantly reduced CFU formation from LSC in a dose-dependent manner but did not inhibit CFU growth from healthy bone marrow cells. The same authors demonstrated the inhibition of LSC in vivo in PDX-AML models, in mice transplanted with MV4-11 and THP-1 cell lines (CDX), and in secondary transplantation models from PDX donor mice treated with NC525. The antileukemic action of NC525 is enhanced in vitro by the presence of collagen, which results in the clustering of LAIR-1 receptors on the LSC, whereas NC525 does not act on healthy stem cells. NanoString RNA expression analysis of human CD45 cells in PDX and CDX mouse models, treated with NC525, showed upregulation of 12 genes and downregulation of 28 genes, including mTOR (2.3-fold), SOS1 (2.3-fold) and BCL-2 (1.8-fold). In vitro protein microarray analysis showed inhibition of phosphorylation of ERK1/2, GSK-3β, and JNK and of the action of AKT, mTORC, and NF-κB by NC525 in LSC but not in healthy ones. Protein expression analysis showed downregulation of the anti-apoptotic protein BCL-XL, suggesting synergy with venetoclax. NC525 demonstrated enhanced killing of cells from AML patients and of MV4-11 cell line in in vitro and in vivo mouse models compared with azacitidine and/or venetoclax monotherapy, with improved efficacy when combined with Azacitidine and/or Venetoclax.

Studies on the MV4-11-LAIR-1^overexpressing^ cell line have also shown that the anti-human IgG monoclonal antibody is able to cause LAIR-1 clustering, even in the absence of collagen, exceeding the threshold required for cell inhibition. The inhibitory activity of NC525 on LSC proliferation is mediated by suppression of the target protein mTORC1 4E-BP1 and an increase in activated caspase-7, but this does not apply to healthy CD34+ cells. The addition of a small-molecule activator of mTOR or a small-molecule inhibitor of caspase-3/7 can inhibit NC525 activity. In vitro and in vivo studies confirm a differential mechanism of NC525, specific for AML cells, in the presence of collagen or of a clustering antibody, which is absent in healthy CD34+ cells. A phase I study with NC525 is ongoing in patients with advanced myeloid neoplasms (NCT05787496).

### 2.7. Immunotherapy

Out of the emerging therapies, CAR-T and BiTEs, which target FLT3 and LSC antigens such as Tim3, CLL-1, CD123, and CD99, seem the most promising options.

CAR-T is composed of three domains: an extracellular signaling domain (SD), derived from a monoclonal antibody directed against a target antigen, a transmembrane, and an intracellular SD.

The efficacy of CAR-T immunotherapy may be limited by immunological escape induced by mutation of genes regulating T-cell recognition and by upregulation of anti-apoptotic genes. The targeting of redundant antigens might cause CAR-T exhaustion and off-target toxicity on normal hematopoietic cells, while that of selective antigens could be susceptible to mechanisms of escape that reduce CAR-T efficacy. Therefore, immunotherapeutic strategies need to be implemented. For these reasons, researchers have developed second- and third-generation CAR-T with one or two costimulatory domains to increase persistence and efficacy. The natural evolution of these therapies is as follows:

− Armored CAR-T, characterized by the addition of a cytokine release mechanism, with potential increased efficacy and toxicity (fourth generation CAR-T);
− Multispecific CAR-T with tandem, split, or inducible antigen recognition, the former characterized by increased efficacy and toxicity, the latter by increased specificity and susceptibility to escape.

**Figure 3:**
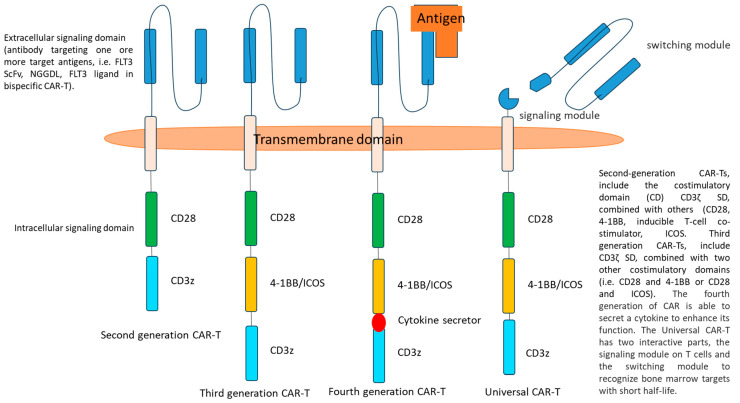
Scheme of ‘old’ and new CAR-T constructs.

Bispecific CAR-T possesses two or different single-chain variable fragments (scFv), capable of recognizing two or more epitopes on the same or different receptors. A hinge region connects the CAR to the transmembrane SD, which links the extracellular with the intracellular SD. In second-generation CAR-T, the costimulatory domain CD3ζ SD, derived from the endogenous T-cell receptor, is combined with others such as CD28 or 4-1BB, OX40, CD27, and inducible T-cell co-stimulator [43]. In third-generation CARs, both CD28 and 4-1BB domains are added as intracellular SDs to improve persistence, proliferation, and efficacy (Figure 3). FLT3L, FLT3, CD33, and CD123 are different antigens targeted by CAR-T, under investigation for the treatment of FLT3 mut AML [44,45,46].

AMG553, a second generation anti-FLT3 CAR-T, including a CD28-CD3z CD, showed a good safety profile in an in vivo model and is currently under investigation in a clinical trial in R/R FLT3mut AML (NCT03904069) [47]. CD123 is the alpha subunit of the interleukin-3 receptor, and its expression on LSC (78% of AMLs, especially FLT3-ITDmut AML) and in R/R AMLs, as well as the low expression by normal hematopoietic progenitors, makes it an ideal target for immunotherapy. CAR-T anti-CD123 demonstrated efficacy in mice xenografted with leukemic cells. These cells produced some results, with low toxicity, 5/7 grade 1-2 CRS, without mortality, in a phase I study in R/R AML and blastic plasmacytoid dendritic cell neoplasm (BPCDN) patients (NCT03190278).

Prolonged hematologic toxicity observed after anti-CD123 CAR T in patients not undergoing subsequent allogeneic hematopoietic cell transplantation motivated researchers to insert on/off switches to turn off acute and long-term side effects.

The universal chimeric antigen receptor (UniCAR) second-generation 2-component platform includes a CD28 costimulatory domain, a first component, which is a CAR that recognizes a targeting module included in the second component, which confers specificity against the tumor antigen of choice. These targeting modules efficiently penetrate bone marrow and solid tumors and have a short half-life of less than 30 min, allowing rapid switching off of the UniCAR system (Figure 3). Preclinical data of anti-CD123 UniCAR suggest that lysis occurs at doses of targeting modules lower than those inducing cytokine release, with better safety, tolerability, and clinical applicability [48].

To minimize the risk of graft-versus-host disease, the CD52 antigen was inserted, in addition to the CD123 antibody, into the signaling domain of allogeneic UNI-CART cells, using Cellectis’ TALEN^®^ technology. This allows the use of Campath to stop the action of the UNI-CARTs, and so modified cells are currently being studied in patients with R/R CD123+ AML patients (AMELI-01, NCT04106076).

To reduce off-target toxicities, Sommer et al. developed a new and promising off-the-shelf, allogeneic anti-FLT3 CAR-R2 T model, which incorporates two rituximab mimotopes (R2) in the extracellular domain, allowing the killing of CAR-T after treatment with Rituximab [49]. In a murine model, rituximab depleted FLT3 CAR T cells, allowing hematological recovery without relapse and providing the rationale for future applications in clinical trials.

Tandem CAR-T, anti-CD123-CD33, CLL61-CD1, and CLL33-CD1 have shown efficacy in mouse models, and the last two are under clinical investigation in R/R AML.

NKG2DL is another antigen overexpressed FLT3mut cell line, MV4-11 and MOLM-13, treated with Gilteritinib. Novel tandem CAR-T, specifically targeting NKG2DL and FLT3 on the AML cell membrane, represents a promising approach with potential synergy with Gilteritinib. FLT3scFv/NKG2D-CAR T cells lysed 7-23% of HSC, but Gilteritinib was not toxic to either CAR-T or normal HSC, while it increased killing of leukemic cell lines in vitro [50].

### 2.8. New Technologies

Innovative immunotherapeutic approaches, including nanoparticles, allogeneic CAR-NK cells, and antibodies directed against FLT3mut AML antigen cells are actually the object of preclinical and clinical investigations.

A new technology of electrostatically complexed nanoparticles allows their contents to be stabilized in vesicles, ensuring their internalization into target cells. Bäumer et al. developed a nanocarrier with the anti-CD33 antibody fraction of Gemtuzumab Ozogamicin, deprived of calicheamicin, cationic protamine, free protamine, siRNA directed against DNMT3A and FLT3ITD mutations and the anionic derivative of the BTK inhibitor ‘Ibrutinib -Cy3.5′ (αCD33-mAB-P/P-DNMT3A-siRNA-Cy5) [51]. These compounds result in self-assembly with IgG chains positioned outside the nanocarrier sphere to facilitate binding to target cells. In contrast, protamines protect the siRNA core by preventing its degradation and facilitating its entry and specific action in mutated leukemic cells in synergy with the BTK inhibitor. Indeed, this nanoparticle efficacy has been demonstrated in vitro, in cell cultures, and in vivo, in xenograft mouse models with both cell lines (DNMT3A-dependent OCI-AML2 and OCI-AML3, FLT3ITD mutated MV4-11) and samples from patients with DNMT3A and FLT3ITDmut AML. Conversely, Bäumer’s nanoparticle does not interfere with the growth of FLT3 and DNMT3Awt cell lines and colonies of normal peripheral blood mononuclear cells.

Researchers have recently engineered allogeneic CAR-NK cells with the aim of reducing the risk of graft vs. host disease, cytokine release, and tumor lysis syndromes, relying on their multiple inhibitory control mechanisms in vivo. In addition, NK cells are not subject to clonal expansion after activation by tumor-specific antigens, which limits their neurological and off-target toxicity. Mansour et al. developed allogeneic FLT3 CAR_sIL-15 NK cells from cord blood NK-92 cell line transduced with lentivirus [52]. The activity of these cells is sustained by the CD28/CD3ζ costimulatory domain, boosted by the release of sIL-15 through a paracrine mechanism, and can be extinguished by cetuximab through the inhibition of the truncated epidermal growth factor receptor inserted into the construct. These CAR-NK cells have demonstrated activity in murine xenograft models with no evidence of limiting toxicity to normal hematopoietic progenitors. Furthermore, the ready availability of an allogeneic product could represent an important tool for reversing a rapidly proliferating disease. Phase I trials are currently investigating the safety of allogeneic peripheral and cord blood CAR-NK cells directed against NKG2D (NCT04623944, NCT05247957), CD123 (cord blood NK, NCT05574608), CD33/CLL1 (peripheral blood NK, NCT05215015) and CD33 (peripheral blood NK, NCT05008575).

The development of antibodies directed against FLT3 represents another interesting challenge for the eradication of FLT3mut LSC. FLT3 is expressed in 80% of AMLs and in B-ALL to a greater extent than CD20 or CD22 antigens. It is hardly lost from leukemic cells, while it is rarely expressed by hematopoietic progenitors. Anti-FLT3 antibodies have shown in vivo efficacy in PDX mouse models. We describe below the characteristics of the latest antiFLT3 antibodies, candidates for use in clinical trials.

Roas et al. [53] identified the anti-FLT3 antibody 20D9 from hybridoma cells of immunized rats and mice as the most specific and active against mutated FLT3 leukemic cell lines (Ba/F3 and MOLM-13) and subsequently conjugated it with the inhibitor of tubulin polymerization, monomethyl auristatine F. The corresponding antibody drug conjugate (ADC) 20D9, thus obtained, confirmed selective efficacy both in murine and human FLT3mut cell lines and in AML-PDX murine model, with irrelevant activity in cultures of normal CD34+ progenitor cells. In addition, kinase inhibitors such as Midostaurin have been shown to increase FLT3 expression and be synergistic with ADC 20D9. These preclinical data represent the rationale for the development of an exciting new immunotherapeutic option, including the anti-FLT3 ADC AG562P1, manufactured by Astellas, currently under investigation in clinical trial NCT02864290.

Mehta et al. developed a bispecific antibody IgG, CLN-049, directed against FLT3 and CD3, targeting AML and B-ALL in a mouse xenograft model, regardless of FLT3 mutation [54], sparing normal hematopoietic cells, making it a possible candidate in future studies.

Phase I clinical trial (NCT02789254) with FLYSYN, an Fc-optimized antibody directed against FLT3/CD135, showed interesting results in eradicating MRD positivity in 35% of 31 patients with AML FLT3 mut [55]. FLYSIN at a single or cumulative dose of 45 mg/m^2^ did not cause dose-limiting toxicity or infusion reactions and resulted in only mild to moderate hematologic and laboratory changes.

## 3. Discussion

FLT3mut LSC might develop resistance to chemotherapy and FLT3i through multiple mechanisms. In this review, we highlighted how recent studies are exploring the combination of multi-targeted drugs that can specifically address these mechanisms, bypassing resistance to apoptosis. Knowledge of the specific metabolism of LSC has contributed to the identification of some new targets susceptible to inhibition. Among these, GSK3 inhibitor BIO and DHODH inhibitors AG636, PTC299, and JNJ-74856665 are some of the new drugs that inhibit the growth of LSC by reducing their metabolism. Studies on the interaction of LSC with the stroma have also highlighted the peculiar role of the monoclonal antibody NC525, an agonist of LAIR-1, currently undergoing a phase I clinical trial. The exploration of the activation pathways of LSC has also led to the identification of other targets and their corresponding inhibitors. Among these, the multikinase inhibitor CG806, the LSD is Bomedemstat and Iademstat, and the AXL/MER tyrosin kinase inhibitor ONO7475, in combination with Venetoclax and/or MCLi and MDM2i, are being studied in clinical trials. Combinations of chemotherapy or hypomethylating agents with FLT3 degraders such as Arsenic and Bortezomib are another attractive approach for overcoming the resistance of FLT3mut LSC. Table 1A shows clinical trials currently recruiting FLT3mut AML patients for treatment with target drugs. 

However, the real challenge, through the future eradication and definitive cure of FLT3mut AML, is immunotherapy. New UNICAR-T and off-the-shelf CAR-T platforms reduce toxicity while preserving specificity and efficacy, enabling the clinical investigation of allogeneic CAR-T with manageable Graft vs. host disease. The use of nanoparticles bound to Immunoglobulin, targeting AML antigens, has demonstrated effective delivery of their contents within AML cells, providing high specificity activity without off-target toxicities and T-cell exhaustion. Anti FLT3 antibodies also represent an intriguing alternative to cell therapy, especially in MRD-positive patients. Table 1B shows all ongoing clinical trials using immunotherapeutic approaches in FLT3mut AML.

Characteristics of all cell lines analyzed in the pre-clinical studies, described in this review, are summarized in Table 2.

## 4. Conclusions

Understanding the biology and mechanisms of resistance of FLT3mut LSC might indicate the new drug targets deserving of future application in clinical trials. Combining MRD-driven approaches with multitarget agents and immunotherapies will hopefully be the future scenario that can address the challenge of treating FLT3mut-resistant AML.

## Figures and Tables

**Table 1:** (**A**) List of studies, registered at ‘clinical trials.gov’, including new target drugs, currently recruiting FLT3mut AML. (**B**) List of studies, registered at clinical trials.gov, including immunotherapy, currently recruiting FLT3mut AML patients.

**(A)**				
**Phase**	**NCT Number**	**ND/RR**	**Drug/Link**	**Sponsor/Link**
I	NCT05947344	RR	STI-8591 (FLT3i) https://clinicaltrials.gov/study/NCT05947344?cond=NCT05947344&rank=1, accessed on 17 March 2024	Zhejiang ACEA
I	NCT05918692	RR	BMF-500 (FLT3i) https://clinicaltrials.gov/study/NCT05918692?cond=NCT05918692&rank=1, accessed on 17 March 2024	Biomea Fusion Inc.
III	NCT05586074	RR	Clifutinib (FLT3i)/LoDAC/Aza https://clinicaltrials.gov/study/NCT05586074?cond=NCT05586074&rank=1, accessed on 17 March 2024	Sunshine Lake
II	NCT05241106	RR	HYML-122 (FLT3i) https://clinicaltrials.gov/study/NCT05241106?cond=NCT05241106&rank=1, accessed on 17 March 2024	Tarapeutics Science Inc.
I/II	NCT05241093	RRt	HYML-122; cytarabine https://clinicaltrials.gov/study/NCT05241093?cond=NCT05241093&rank=1, accessed on 17 March 2024	Tarapeutics Science Inc.
III	NCT04716114	RR	SKLB1028 (EGFR-FLT3-Abli) vs. CHT https://clinicaltrials.gov/study/NCT04716114?cond=NCT04716114&rank=1, accessed on 17 March 2024	CSPC ZhongQi
I/II	NCT03922100	RR	NMS-03592088 (FLT3-KIT-CSF1Ri) https://clinicaltrials.gov/study/NCT03922100?cond=NCT03922100&rank=1, accessed on 17 March 2024	Nerviano Medical Sciences
II	NCT05199051	RR	GO/ID Cytarabine/Gilteritinib https://clinicaltrials.gov/study/NCT05199051?cond=NCT05199051&rank=1, accessed on 17 March 2024	Centre Antoine Lacassagne
I	NCT05024552	RR	Gilteritinib/CPX-351 https://clinicaltrials.gov/study/NCT05024552?cond=NCT05024552&rank=1, accessed on 17 March 2024	H. Lee Moffitt CCR Institute
I	NCT04278768	RR	Emavusertib (IRAK4i)/Ven https://clinicaltrials.gov/study/NCT04278768?cond=NCT04278768&rank=1, accessed on 17 March 2024	Curis, Inc.
I	NCT05546580	RR	Iadademstat (LSD1i)/Gilteritinib https://clinicaltrials.gov/study/NCT05546580?cond=NCT05546580&rank=1, accessed on 17 March 2024	Oryzon Genomics S.A.
I	NCT05597306	RR	Bomedemstat (LSD1i)/Ven https://clinicaltrials.gov/study/NCT05597306?cond=NCT05597306&rank=1, accessed on 17 March 2024	Terrence J Bradley, MD
I	NCT03900949	ND	Midostaurin/Cytarabin/Daunorubicin/GO https://clinicaltrials.gov/study/NCT03900949?cond=NCT03900949&rank=1, accessed on 17 March 2024	Uma Borate
I/II	NCT05520567	ND	Gilteritinib/Ven/Aza https://clinicaltrials.gov/study/NCT05520567?cond=NCT05520567&rank=1, accessed on 17 March 2024	Astellas Pharma
I/II	NCT05010122	RR/ND	Decitabine-Cedazuridine/Gilteritinib/Ven https://clinicaltrials.gov/study/NCT05010122?cond=NCT05010122&rank=1, accessed on 17 March 2024	M.D.A.C.C
I/II	NCT04385290	ND	Midostaurin/Cytarabin/Daunorubicin/GO https://clinicaltrials.gov/study/NCT04385290?cond=NCT04385290&rank=1, accessed on 17 March 2024	Universität Dresden
(**B**)				
**Phase**	**NCT Number**	**AML ND/RR**	**Immunotherapy/** **Link**	**Sponsor**
I	NCT06201247	RR CD123pos	Off-the-shelf anti-CD123 CAR-NK https://clinicaltrials.gov/study/NCT06201247?cond=NCT06201247&rank=1, accessed on 17 March 2024	Peking University
I	NCT06006403	RR CD123pos	Anti-CD123 CAR-NK cells https://clinicaltrials.gov/study/NCT06006403?cond=NCT06006403&rank=1, accessed on 17 March 2024	Chongqing Precision Biotech Co., Ltd.
I	NCT05574608	RR CD123pos	Anti-CD123-CAR-NK cells https://clinicaltrials.gov/study/NCT05574608?cond=NCT05574608&rank=1, accessed on 17 March 2024	Academy of MilitaryMedical Sciences
I	NCT03190278	RR CD123pos	Allo UCART anti-123v1.2 https://clinicaltrials.gov/study/NCT03190278?cond=NCT03190278&rank=1, accessed on 17 March 2024	Cellectis S.A.
I	NCT05995041	RR AML	Anti-CLL-1, CD33, CD38 CD123 UCAR-T https://clinicaltrials.gov/study/NCT05995041?cond=NCT05995041&rank=1, accessed on 17 March 2024	Shenzhen Geno-Immune Medical Institute
I	NCT05984199	RR CD33pos	VCAR33, anti-CD33 CAR-T https://clinicaltrials.gov/study/NCT05984199?cond=NCT05984199&rank=1, accessed on 17 March 2024	Vor Biopharma
I	NCT05945849	RR CD33pos	CD33KO-HSPC; anti-CD33 CAR-T https://clinicaltrials.gov/study/NCT05945849?cond=NCT05945849&rank=1, accessed on 17 March 2024	University of Pennsylvania
I	NCT05672147	RR CD33pos	Anti-CD33 CAR T https://clinicaltrials.gov/study/NCT05672147?cond=NCT05672147&rank=1, accessed on 17 March 2024	City of Hope Medical Center
I	NCT05105152	RR CD33pos	SC-DARIC33 CD33 CAR-T https://clinicaltrials.gov/study/NCT05105152?cond=NCT05105152&rank=1, accessed on 17 March 2024	Seattle Children’s Hospital
I	NCT05665075	RR CD33pos	QN-023°anti-CD33 NK https://clinicaltrials.gov/study/NCT05665075?cond=NCT05665075&rank=1, accessed on 17 March 2024	Zhejiang University
I/Ib	NCT03927261	RR CD33 pos	PRGN-3006 anti-CD33 CAR-T https://clinicaltrials.gov/study/NCT03927261?cond=NCT03927261&rank=1, accessed on 17 March 2024	Precigen, Inc
I	NCT05445011	RR FLT3mut	TAA05 anti-FLT3 CAR-T https://clinicaltrials.gov/study/NCT05445011?cond=NCT05445011&rank=1, accessed on 17 March 2024	Wuhan Union Hospital, China
I	NCT05432401	RR FLT3mut	TAA05 anti-FLT3 CAR-T https://clinicaltrials.gov/study/NCT05432401?cond=NCT05432401&rank=1, accessed on 17 March 2024	PersonGen BioTherapeutics (Suzhou) Co., Ltd.
I	NCT05023707	RR FLT3mut	Anti-FLT3 CAR-T https://clinicaltrials.gov/study/NCT05023707?cond=NCT05023707&rank=1, accessed on 17 March 2024	The First Affiliated Hospital of Soochow University
I	NCT05143996	RR FLT3mut	CLN-049, Antibody anti FLT3/CD3 iv/sc https://clinicaltrials.gov/study/NCT05143996?cond=NCT05143996&rank=1, accessed on 17 March 2024	Cullinan Oncology Inc.
I	NCT04623944	RR	NKX101, allogeneic antiNKG2DL CAR NK https://clinicaltrials.gov/study/NCT04623944?cond=NCT04623944&rank=1, accessed on 17 March 2024	Nkarta Inc.
I	NCT05734898	RR	NKG2D CAR-NK https://clinicaltrials.gov/study/NCT05734898?cond=NCT05734898&rank=1, accessed on 17 March 2024	Zhejiang University

RR: relapse refractory, ND: newly diagnosed, LoDAC: low dose cytarabine, Aza: azacitidine, ID: intermediate dose, Ven: Venetoclax, GO: Gemtuzumab Ozogamicin.

**Table 2:** Description of characteristics of cell lines analyzed in the pre-clinical studies reported in the review.

Name	Characteristics	References in the Text
Kasumi-1	AML, M2, t(8;21), relapse	[5]
HL-60	AML, M3	[5]
MV4-11	AML, M5, t(4;11), FLT3ITDmut	[8,24,47,48]
THP-1	AML, M4, t(9;11) (p21;q23), (MLL-AF9; MLL-MLLT3)	[24]
MOLM-13	AML M5a, FLT3ITDmut	[47,50]
OCI-AML2	AML, M4, DNMT3Amut R635W	[48]
OCI-AML3	AML, M4, NPM1mut (type A), DNMT3Amut R882C	[48]
NK-92	NK lymphoma	[49]
Ba/F3	IL-3-dependent murine pro B cell line, FLT3ITDmut	[50]

## Abbreviations

ADC	Antibody drug conjugate.
AXL	Anexelekto.
BiTEs	Bispecific T cell engager molecules.
CAR-T	Chimeric antigen receptor T cells.
DHODH	Dihydroorotate dehydrogenase.
FLT3i	FLT3 inhibitors.
FLT3mut	FLT3 mutated AMLs.
GSK3	Glycogen synthase kinase 3.
HSC	Hematopoietic stem cell.
H3K9	Hystone 3 Lysin 9.
IGF2	Insulin-like growth factor 2.
LAIR-1	Leukocyte-associated immunoglobulin-like receptor-1.
LSC	Leukemic stem cell.
LSD1	lysine-specific demethylase 1.
MRD	Minimal residual disease.
NKGD2L	Natural killer group 2 member D ligand.
PDX	Patient-derived xenograft.
RR	Relapse refractory.
SD	Signaling domain.
UniCAR	Universal chimeric antigen receptor.
